# Health and economic outcomes of a universal early intervention for parents and children from birth to age five: evaluation of the Salut Programme using a natural experiment

**DOI:** 10.1186/s12962-023-00439-7

**Published:** 2023-05-04

**Authors:** Filipa Sampaio, Jenny Häggström, Richard Ssegonja, Eva Eurenius, Anneli Ivarsson, Anni-Maria Pulkki-Brännström, Inna Feldman

**Affiliations:** 1 Department of Public Health and Caring Sciences, Uppsala University, BMC, Husargatan 3, Uppsala, 751 22 Sweden; 2 Department of Statistics, Umeå School of Business and Economics, Umeå University, Umeå, Sweden; 3 Department of Medical Sciences, Respiratory-, Allergy- and Sleep Medicine Research Unit, Uppsala University, Uppsala, Sweden; 4 Department of Epidemiology and Global Health, Umeå University, Umeå, Sweden

**Keywords:** Early intervention, Universal prevention, Child health, Maternal health costs, Health care costs

## Abstract

**Background:**

The aim of this study was to investigate the health and economic outcomes of a universal early intervention for parents and children, the Salut Programme, from birth to when the child completed five years of age.

**Methods:**

This study adopted a retrospective observational design using routinely collected linked register data with respect to both exposures and outcomes from Västerbotten county, in northern Sweden. Making use of a natural experiment, areas that received care-as-usual (non-Salut area) were compared to areas where the Programme was implemented after 2006 (Salut area) in terms of: (i) health outcomes, healthcare resource use and costs around pregnancy, delivery and birth, and (ii) healthcare resource use and related costs, as well as costs of care of sick child. We estimated total cumulative costs related to inpatient and specialised outpatient care for mothers and children, and financial benefits paid to mothers to stay home from work to care for a sick child. Two analyses were conducted: a matched difference-in difference analysis using the total sample and an analysis including a longitudinal subsample.

**Results:**

The longitudinal analysis on mothers who gave birth in both pre- and post-measure periods showed that mothers exposed to the Programme had on average 6% (95% CI 3–9%) more full-term pregnancies and 2% (95% CI 0.03-3%) more babies with a birth weight ≥ 2500 g, compared to mothers who had care-as-usual. Savings were incurred in terms of outpatient care costs for children of mothers in the Salut area ($826). The difference-in-difference analysis using the total sample did not result in any significant differences in health outcomes or cumulative resource use over time.

**Conclusions:**

The Salut Programme achieved health gains, as a health promotion early intervention for children and parents, in terms of more full-term pregnancies and more babies with a birth weight ≥ 2500 g, at reasonable cost, and may lead to lower usage of outpatient care. Other indicators point towards positive effects, but the small sample size may have led to underestimation of true differences.

**Supplementary Information:**

The online version contains supplementary material available at 10.1186/s12962-023-00439-7.

## Background

Prenatal, infancy and childhood periods constitute the most sensitive phases in a person’s development. The relationship between early-life health and long-term health and economic outcomes is widely known [1–4], thus, early interventions targeting the promotion of healthy behaviors and the prevention of risk factors could be particularly effective [5]. As a result, health promotion during pregnancy and early childhood has become an important public health concern.

As a society, we are interested in maximizing the potential of early interventions because the potential health and economic benefits of reducing avoidable ill health are well documented [6–8]. While evidence on the effectiveness and cost-effectiveness of some early interventions exists [9–11], evidence on the economic benefits of such interventions targeting the periods of pregnancy and early childhood is limited. A recently published review of existing early childhood interventions reported that most programs have favorable effects on at least one child outcome, and those with an economic evaluation reported favorable economic returns [7]. This review also highlighted that only few programs that began in the prenatal period had been rigorously evaluated, used large sample sizes or had sufficiently long follow-ups. Evidence on both the health and economic impacts of interventions is essential to support decision makers in the process of allocation of societal resources [12].

An evaluation of a well-known nurse-led intensive home visiting programme for first-time teenage mothers (Family Nurse Partnership, FNP) in the United Kingdom found that it yielded large costs and only small benefits at a two-year follow-up [13]. One of the reasons behind these results might be a relatively short follow-up period. Given that early childhood interventions are hypothesized to have their main effects beyond their usually short follow-up periods, it is important to identify and include the broader and longer-term impacts associated with such complex interventions. Potential benefits could be observed later on, because of improvements in primary outcomes of the Family Nurse Partnership – intervention, by revealing associations between maternal risk factors and longer term child outcomes [14]. It is, thus, fundamental to consider the broader and longer-term impacts of early interventions for both parents and their children when conducting evaluation exercises. Several studies have estimated the lifetime costs related to preterm birth and they represent the potential economic gains from interventions that can improve such outcomes. Waitzman et al. (2016) [15] estimated the excess costs of prematurity for a cohort of babies born 2016 in the US to be $25.2 billion: $17.1 billion for medical care of the preterm babies, $2.0 billion for delivery care, $1.3 billion for early intervention and special education, and $4.8 billion in productivity losses due to disability in adult age (2016 prices).

In 2006, the Swedish Salut Child Health Promotion Programme (Salut Programme) was piloted and gradually rolled out in the Västerbotten county in Northern Sweden targeting all expectant parents and children up to age 5 years [16]. It offered, in addition to care-as-usual (CAU), multisectorial efforts to promote parental and child health, including a comprehensive package of interventions using a family-centred approach, and being integrated into routine practises to reach all. Both care-as-usual and the Salut Programme are provided mainly by antenatal and child healthcare services, but dental services and open pre-schools are also involved. All are free of charge for the parents. Some of the Programme’s aims are to prevent maternal and child pregnancy complications related to maternal lifestyles, as well as to support prevention activities in a variety of sectors concerning mental health and healthy lifestyles of expectant parents, and later, their children. The Programme includes age-specific modules, and starts with the first module during pregnancy and, according to the age of the child, continues with specific interventions [17]. All interventions target different topics related to pregnancy and birth, for expectant parents, and topics related to the stage of development of the child. Continuous efforts are being made to keep up sustainability over time and develop the Programme to be up-to-date as evidence develop. For example, all professionals involved are invited, on a regular basis, to educational seminars, and programme manuals are available to guide practice (see table S1 in the supplementary appendix). The Salut Programme is described in detail elsewhere [18, 19].

In a previous evaluation, we have investigated the short-term effectiveness and cost-effectiveness of the Salut Programme over the periods of pregnancy, delivery and the child’s first two years of life, based on register data. The study found that the Programme improved average health outcomes at birth (positive improvement in Apgar scores at 1 and 5 min, reflecting the child’s physical condition), at lower costs than CAU, with a 50% probability of representing good value for money [18]. After the evaluation, data on health outcomes and resource use for these mothers and children has continuously been collected via national registers. Further analyses on the longer-term impacts of the Programme on maternal and child outcomes and resource use are warranted.

The overall aim of the current paper was to investigate the health and economic outcomes of the Salut Programme, a universal early intervention for parents and children, up to when the child completed five years of age. The specific objectives were to investigate the differential impact of the Programme, compared with care-as-usual, on: (i) a set of health outcomes around pregnancy, delivery and birth; and (ii) cumulative healthcare resource use and related costs, as well as costs related to care of sick child, from birth to when the child completed five years of age.

## Methods

### Study design and participants

This study adopted a retrospective observational design using routinely collected register data with respect to both exposures and outcomes. This data, collected independently of our study, allowed us to simulate an experiment by taking advantage of the stepwise implementation of the Salut Programme in the Västerbotten county. We were able to identify areas that received care-as-usual (non-Salut area) and areas where the Programme was implemented after 2006 (Salut area). Inclusion of mothers in either group was based on the place of residence at the time of childbirth. Compared to the first evaluation of the Salut Programme [18], in this present study, the pre-measure groups were redefined to make sure individuals born before the Programme was implemented did not receive some of the interventions for older children. Mothers and their children from both the Salut and non-Salut areas were included if: (1) the child was born 2000–2002 (pre-measure period) or (2) the child was born 2006–2008 (post-measure period). Four groups were formed for analysis: Salut pre, Salut post, non-Salut pre and non-Salut post. Groups receiving the Programme were compared with groups receiving care-as-usual in terms of: (i) health outcomes, healthcare resource use and related costs around the periods of pregnancy, delivery and birth, and (ii) healthcare resource use and related costs, as well as costs related to care of sick children, around delivery and birth up until the child completed five years of age.

**Fig. 1:**
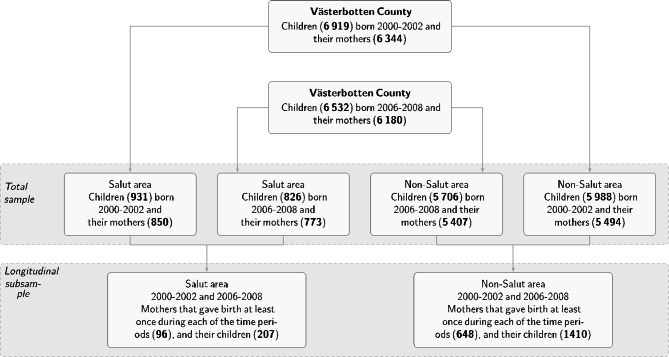
An overview of the study population and samples used in the analyses

Figure 1 provides an overview of the study population and the samples used in the analyses.

### Data sources

Data from 2000 to 2014 was retrieved from several national registers and linked on an individual level by Statistics Sweden. Intergenerational links between parents and children were created by using individual personal identification numbers (ID) available for all Swedish residents. In the current study, data from six national registers were included, namely: (i) The total population register, including demographic data; (ii) The Multi-Generation Register, including generational links; (iii) The Register of Education, including information on the population’s educational level; (iv) The Medical Birth Register, including data on mothers pregnancy and delivery and newborns; (v) The National Patient Register, including inpatient care and specialized outpatient care data; and (vi) The Income and taxation register, including data on financial benefits related to care of sick child. Further information on the data sources used is described elsewhere [19].

### Health outcomes and resource use

Health outcomes for mothers and their children were chosen according to the aims of the Salut Programme and its expected impacts on mothers and children’s health and wellbeing, as well as according to the data available in the registers.

The following health outcomes around pregnancy, delivery and birth for both mothers and children were considered for the present study: mother’s smoking status at first antenatal visit (yes/no); pregnancy length at delivery (≥ 37/<37 weeks); caesarean section (yes/no); birth weight (≥ 2500/<2500 g); birth length (cm); large for gestational age (≥ 2 SD above the reference population’s mean weight); small for gestational age (≤ 2 SD below the reference population’s mean weight); Apgar score 1, 5 and 10 min after delivery (≥ 7/<7 points) (reflecting obstetric care, but also the obstetric process overall); and child diagnosed by paediatrician as healthy (yes/no).

The following health care resource use for mothers and children around delivery and birth up until the child completed five years of age were considered: duration of mother’s inpatient care related to delivery (days), cumulative duration of inpatient care for mothers and children (days), cumulative duration of day patient and specialized outpatient visits for mothers and children.

### Costs

Costs were estimated for inpatient care and specialised outpatient care for mother and children, respectively, for the period from birth to when the child completed five years of age. Total costs were estimated for different age-cohorts of children and their mothers, according to year of birth and year of completion of five years of age. Children were followed up during seven years. Thus, all data for children born 2000–2002 before Salut implementation were clustered in three groups: 2000–2006, 2001–2007, 2002–2008. Data for children born 2006–2008 after Salut implementation were clustered as: 2006–2012, 2007–2013, 2008–2014, hence the costing period spanned 2000–2014.

Inpatient and outpatient care costs for mothers and children were the product of resource frequencies (number of inpatient days and number of outpatient visits) by the average cost per inpatient day and average cost per outpatient visit, respectively. Average costs were retrieved from the Cost per Patient database (KPP) held by the Swedish Association of Local Authorities and Regions [20]. The KPP database contains information about which healthcare was provided to patients, by whom, to what type of patients and what resources were used in each healthcare contact. The KPP estimates are based on the total production cost of healthcare, i.e. the gross cost incurred to deliver healthcare. Average unit costs were only available from 2004 for inpatient care and 2008 for outpatient care, thus linear prediction models were used.to estimate the data for full period, 2000–2014. Unit costs used in the costing analysis are presented in Table S2 in the supplementary appendix.

Total cumulative financial benefits paid to mothers to stay home from work to care for a sick child were also estimated. Healthcare costs as well as costs related to care of sick child were aggregated for the different age-cohorts from birth to when the child completed five years of age.

Costs were uprated to 2020 Swedish Krona using inflation indices [21] and converted to $US 2020 prices using purchasing power parities [22].

### Data analysis

Two analyses were conducted: a matched difference-in difference analysis using the total sample and an analysis including a longitudinal subsample.

In the matched difference-in-difference analysis, each mother to a child born in the Salut area in the post-measure period (Salut post) was matched to a mother to a child born in each of the other groups, Salut pre, non-Salut pre and non-Salut post. Mothers were matched on the mother’s age and educational level at the time of the child’s birth, in each group. For every outcome assessed, an observation was considered a match if the mother, at the time of the child’s birth, had the same level of education and similar age as the mother of a child born in the Salut area at post measure. The means for each group were used to calculate a difference-in-difference estimate of the average treatment effect on the treated (ATT). This estimate was computed by subtracting the average difference over time in the non-Salut area from the average difference over time in the Salut area. Confidence intervals were computed using the standard errors (SE) based on non-parametric bootstrapping with 1000 replications to reflect the uncertainty around the ATT point estimate. P-values were estimated assuming a normal distribution of the ATT point estimate.

The longitudinal analysis included only a sub-sample of mothers who gave birth in both the pre- and post-measure periods and remained living in the same geographical area over the full analysis period. Using this sub-sample made it possible to use the mother´s premeasure outcome value as a covariate to match on. As such, for each outcome of interest (health outcomes, resource use and costs), mothers were matched on the outcome value at pre-measure in addition to the matching variables used in the difference-in-difference analysis (mother’s age and educational level). The intervention estimate of ATT was computed by subtracting the average outcome in the non-Salut area at post-measure from the average outcome in the matched participants in the Salut area at post-measure. Matching was performed separately for each outcome. A Bonferroni correction was applied to account for multiple comparisons. Confidence intervals were computed using Abadie-Imbens SE [23] to reflect the uncertainty around the ATT estimates. These SEs are based on the asymptotic variance of the simple matching estimator and are preferred to bootstrapping to avoid inconsistent SE estimation [24]. Analysis were conducted in R version 3.6.2 using the Matching package for matching and Abadie-Imbens SE.

This study assesses a range of outcomes for both children and mothers, thus a cost-consequence analysis framework is relevant to inform decision-makers where costs and outcomes of an intervention fall on different domains [25]. All costs and outcomes are therefore presented in a descriptive and disaggregated way, where no estimates of cost-effectiveness (i.e. incremental cost effectiveness ratios) were computed but rather incremental costs and outcomes per mother and child for Salut and non-Salut arms are presented for each cost and outcome item.

## Results

### Study population

Characteristics of the mothers and their children in each geographical area at pre- and post-measures are reported for the total sample (Table 1) and the longitudinal subsample (Table 2). For the total sample, in the Salut area, 850 mothers and 931 children were included at pre-measure, and 773 mothers and 826 children were included at post-measure. In the non-Salut area, 5,494 mothers and 5,988 children were included at pre-measure, and 5,407 mothers and 5,706 children were included at post-measure. For the longitudinal sample, in the Salut area, 96 mothers and 107 children were included at pre-measure, and 96 mothers and 100 children were included at post-measure. In the non-Salut area, 648 mothers and 736 children were included at pre-measure, and 648 mothers and 674 children were included at post-measure. In both samples, mothers were on average younger and less educated in the Salut area compared to the non-Salut area. For the total sample, the difference in mothers’ age between Salut post and non-Salut post was statistically significant (p-value 0.007). Conversely, between non-Salut pre- and non-Salut post, and between Salut pre and Salut post, there were no significant differences in mothers’ age (p-values 0.17 and 0.19, respectively). The differences in mothers’ education between Salut post and each of the other three groups were all statistically significant with p-values below 0.01. Missing values varied between measures. Information on mother’s education was missing for 1.3–2.7% of the Salut area observations and 0.8–0.9% of the non-Salut area observations. Most outcomes at birth exhibited some missingness, with the largest proportion for the smoking variable (24% in non-Salut pre).

**Table 1:** Characteristics of the participants in the total sample

	Salut area^a^		Non-Salut area^a^	
	pre^b^	post^b^	pre^b^	post^b^
**Participants**				
Mothers, n	850	773	5,494	5,407
Children, n	931	826	5,988	5,706
**Covariates**				
Mother’s age (years), M (SD)	29.0 (5.0)	29.3 (5.2)	29.6 (4.8)	30.0 (5.0)
Mother’s education, %				
Compulsory school	8.5	10.9	7.6	7.1
Secondary school	57.2	48.8	49.3	36.6
Higher education	34.3	40.2	43.1	56.3
**Health outcomes**				
***Pregnancy, delivery and around the child’s birth***				
Smoking^+ c^ (yes), %	7.6	4.6	5.8	3.8
Pregnancy length^+^ (≥ 37 weeks), %	93.9	95.5	94.3	94.6
Caesarean section^+^ (yes), %	18.0	18.0	15.8	16.4
Birth weight^++^ (≥ 2 500 g), %	94.9	97.2	96.1	96.5
Birth length^++^ (cm), M (SD)	50.3 (2.7)	50.3 (2.6)	50.5 (2.6)	50.3 (2.5)
LGA^++d^ (yes), %	3.1	3.8	4.2	3.3
SGA^++e^ (yes), %	1.9	2.5	1.9	1.8
Apgar score^++f^ (≥ 7 points) at 1 min, %	95.6	96.2	94.8	94.6
at 5 min, %	99.0	99.5	98.6	98.3
at 10 min, %	99.5	99.8	99.5	99.5
Healthy child^++g^ (yes), %	79.2	81.2	78.5	79.1
Mother’s inpatient care^+ h^ (days), M (SD)	2.3 (1.8)	2.6 (1.5)	2.2 (1.8)	2.4 (1.5)
***From birth to age 5***				
Mother’s inpatient care^j +^ (days), M (SD)	1.8 (8.7)	2.1 (14.9)	1.5 (12.8)	1.4 (14.0)
Child’s inpatient care^j ++^ (days), M (SD)	2.6 (8.3)	2.2 (13.0)	3.1 (1.4)	2.0 (1.3)
Mother’s day patient visits^k+^, M (SD)	0.0 (0.2)	0.1 (0.3)	0.0 (0.3)	0.0 (0.2)
Child’s day patient visits^k++^, M (SD)	0.1 (0.8)	0.1 (0.7)	0.1 (0.6)	0.1 (0.9)
***From age 1 to 5***				
Mother’s specialized outpatient doctor’s visits+^l^	2.2 (4.1)	5.1 (7.3)	2.2 (4.1)	4.4 (6.5)
Child’s specialized outpatient doctor’s visits++^l^	2.2 (3.6)	6.1 (7.8)	2.4 (3.9)	6.2 (8.1)

M – mean; SD – Standard deviation.

^a^ Salut area – Geographical area in Västerbotten county where the Salut Programme was implemented from 2006 and onwards; non-Salut area – remaining part of Västerbotten county.

^b^ Premeasure period 2000–2002; postmeasure period 2006–2008

^++^ Outcome child health; + Outcome maternal health

^c^ Smoking status at first antenatal visit, around pregnancy week 12.

^d^ Large for gestational age (LGA) – ≥2 SD above the reference population’s mean weight.

^e^ Small for gestational age (SGA) – ≤2 SD below the reference population’s mean weight.

^f^ A measure of the newborn’s physical condition 1, 5 and 10 min after birth, range 0–10.

^g^ A healthy child according to a paediatrician’s examination.

^h^ Mother’s inpatient care related to delivery.

^i^ Early inpatient care for mother and child, respectively, during first two months after the child’s birth (not related to delivery).

^j^ Cumulative duration of inpatient care for mother and child, respectively, over the child’s first five years, excluding care due to delivery complications.

^k^ Number of day patient visits for mother and child, respectively, over the child’s first five years, excluding care for the mother due to delivery complications.

^l^ Number of specialized outpatient doctor’s visits for mother and child, respectively, over the time when child is between one and five years old, excluding care for the mother due to delivery complications.

**Table 2:** Characteristics of the participants in the longitudinal subsample

	Salut area^a^		Non-Salut area^a^		
	**pre** ^b^	**post** ^b^	**pre** ^b^	**post** ^b^	
**Participants**					
Mothers, n	96	96	648	648	
Children, n	107	100	736	674	
**Covariates**					
Mother’s age (years), M (SD)	26.1 (3.8)	31.9 (3.8)	26.8 (4.0)	32.7 (4.0)	
Mother’s education, %					
Compulsory school	6.1	9.0	10.5	8.0	
Secondary school	65.7	57.0	51.0	46.7	
Higher education	28.3	34.0	38.5	45.2	
**Health outcomes**					
***Pregnancy, delivery and around the child’s birth***					
Smoking^c+^ (yes), %	7.4	3.1	4.9	5.0	
Pregnancy length+ (≥ 37 weeks), %	90.7	100.0	94.4	93.8	
Caesarean section+ (yes), %	14.0	15.0	14.4	18.5	
Birth weight++ (≥ 2 500 g), %	94.4	100.0	95.9	97.3	
Birth length++ (cm), M (SD)	49.7 (3.7)	50.5 (2.0)	50.6 (2.7)	50.6 (2.3)	
LGA++^d^ (yes), %	4.7	3.0	3.4	5.2	
SGA++^e^ (yes), %	1.9	1.0	2.2	1.3	
Apgar score++^f^ (≥ 7 points) at 1 min, %	95.3	100.0	93.5	96.0	
at 5 min, %	98.1	100.0	98.2	98.7	
at 10 min, %	99.0	99.0	99.7	99.8	
Healthy child++^g^ (yes), %	81.3	87.0	78.1	82.0	
Mother’s inpatient care+^h^ (days), M (SD)	2.1 (1.8)	2.2 (1.3)	2.3 (1.8)	2.1 (1.5)	
***From birth to age 5***					
Mother’s inpatient care^j ++^ (days)	0.7 (2.4)	1.9 (7.3)	1.5 (12.6)	1.7 (7.9)	
Child’s inpatient care^j +^ (days)	2.9 (8.1)	1.0 (2.2)	3.1 (13.1)	2.9 (18.1)	
Mother’s day patient visits^k++^	0.0 (0.2	0.0 (0.2)	0.0 (0.2)	0.1 (0.2)	
Child’s day patient visits^k+^	0.1 (0.3)	0.1 (0.2)	0.1 (0.4)	0.2 (2.6)	
***From age 1 to 5***					
Mother’s specialized outpatient doctor’s visits^l+^	1.9 (2.6)	4.3 (4.2)	1.9 (3.3)	4.8 (7.3)	
Child’s specialized outpatient doctor’s visits^l++^	2.7 (5.8)	4.9 (6.0)	2.4 (3.6)	6.3 (9.0)	

M – mean; SD – Standard deviation. + Outcome maternal health; ++ Outcome child health

^a^ Salut area – Geographical area in Västerbotten county where the Salut Programme was implemented from 2006 and onwards; non-Salut area – remaining part of Västerbotten county.

^b^ Premeasure period 2000–2002; postmeasure period 2006–2008.

^c^ Smoking status at first antenatal visit, around pregnancy week 12.

^d^ Large for gestational age (LGA) – ≥2 SD above the reference population’s mean weight.

^e^ Small for gestational age (SGA) – ≤2 SD below the reference population’s mean weight.

^f^ A measure of the newborn’s physical condition 1, 5 and 10 min after birth, range 0–10 points.

^g^ A healthy child according to a paediatrician’s examination.

^h^ Mother’s inpatient care related to delivery.

^i^ Early inpatient care for mother and child, respectively, during the first two months after the child’s birth (not related to delivery).

^j^ Cumulative duration of inpatient care for mother and child, respectively, over the child’s first five years, excluding care due to delivery complications.

^k^ Number of day patient visits for mother and child, respectively, over the child’s first five years, excluding care for the mother due to delivery complications.

^l^ Number of specialized outpatient doctor’s visits for mother and child, respectively, over the time when child is between one and five years old, excluding care for the mother due to delivery complications.

### Health outcomes and resource use

Samples were slightly unbalanced before matching and the covariate balance was improved with matching. Sample sizes differed per outcome as matching was done independently for each outcome. Table 3 shows the differences in health outcomes and resource use between Salut and non-Salut areas, for the total sample and the longitudinal sample.

**Table 3:** Differences in health outcomes and resource use between Salut and non-Salut areas, for the total sample and the longitudinal subsample

	Total sample		Longitudinal subsample	
Health outcomes	ATT (95% CI)^a^	p-value	ATT (95% CI)^b^	p-value
Pregnancy, delivery and around the child’s birth				
Smoking^c^ (yes)^+^	-0.02 (-0.05, 0.01)	0.32	-0.01 (-0.06, 0.03)	0.51
Pregnancy length (≥ 37 weeks) ^+^	0.01 (-0.01, 0.04)	0.36	0.06 (0.03, 0.09)	7e-05**
Caesarean section (yes) ^+^	-0.02 (-0.06, 0.03)	0.41	0.01 (-0.07, 0.08)	0.87
Birth weight (≥ 2500 g) ^++^	0.02 (-3e-04, 0.04)	0.05	0.02 (9e-03, 0.03)	4e-04*
Birth length (cm) ^++^	0.22 (-0.07, 0.51)	0.14	-0.14 (-0.59, 0.32)	0.56
LGA^d^ (yes) ^++^	0.01 (-0.01, 0.03)	0.46	-0.03 (-0.08, 0.02)	0.21
SGA^e^ (yes) ^++^	0.01 (-0.01, 0.02)	0.31	4e-03 (-0.02, 0.03)	0.74
Apgar score^f + +^ (≥ 7 points) at 1 min	0.01 (-0.02, 0.03)	0.61	0.02 (0.01, 0.04)	3e-03
at 5 min	0.01 (-4e-03, 0.02)	0.25	0.01 (-5e-05, 0.01)	0.05
at 10 min	2e-03 (-0.01, 0.01)	0.62	-0.01 (-0.03, 0.01)	0.39
Healthy child^g + +^ (yes)	0.04 (-0.01, 0.08)	0.11	0.03 (-0.05, 0.12)	0.46
Mother’s inpatient care^h + +^ (days)	0.07 (-0.11, 0.26)	0.44	0.21 (-0.17, 0.58)	0.27
**From birth to 5**				
Mother’s inpatient care^j ++^ (days)	-0.24 (-1.40, 0.91)	0.68	0.94 (-0.81, 2.67)	0.29
Child’s inpatient care^j +^ (days)	0.80 (-0.55, 2.15)	0.25	-1.17 (-2.76, 0.42)	0.15
Mother’s day patient visits^k++^	0.01 (-0.01, 0.04)	0.33	-0.03 (-0.09, 0.02)	0.27
Child’s day patient visits^k+^	0.01 (-0.06, 0.08)	0.78	-0.02 (-0.07, 0.04)	0.58
**From age 1 to 5**				
Mother’s specialized outpatient doctor’s visits^l+^	0.34 (-0.31, 0.98)	0.31	-1.64 (-3.72, 0.44)	0.12
Child’s specialized outpatient doctor’s visits^l++^	0.08 (-0.58, 0.75)	0.81	-1.77 (-3.09, -0.44)	9e-03

^+^ Outcome maternal health

^++^ Outcome child health

^a^ Difference-in-difference estimates of the average treatment effect on the treated (ATT) with 95% confidence intervals (CI). CIs and p-values were computed with the assumption that ATT was normally distributed and with a standard deviation equal to the bootstrap standard error.

^b^ Simple matching estimates of the average treatment effect on the treated (ATT) with 95% confidence intervals (CI).

CIs and p-values were computed with the assumption that ATT was normal distributed and with a standard deviation equal to the Abadie-Imbens standard error.

^c^ Smoking status at first antenatal visit, around pregnancy week 12.

^d^ Large for gestational age (LGA) – ≥2 SD above the reference population’s mean weight.

^e^ Small for gestational age (SGA) – ≤2 SD below the reference population’s mean weight.

^f^ A measure of the newborn’s physical condition 1, 5 and 10 min after birth, range 0–10.

^g^ A healthy child according to a paediatrician’s examination.

^h^ Mother’s inpatient care related to delivery.

^i^ Early inpatient care for mother and child, respectively, during the first two months after the child’s birth but not related to the delivery.

^j^ Cumulative duration of inpatient care for mother and child, respectively, over the child’s first five years, excluding care due to delivery complications.

^k^ Number of day patient visits for mother and child, respectively, over the child’s first five years, excluding care for the mother due to delivery complications.

^l^ Number of specialized outpatient doctor’s visits for mother and child, respectively, over the time when child is between one and five years old, excluding care for the mother due to delivery complications.

*Statistically significant effect at the α = 0.05 level after a Bonferroni correction for multiple comparisons, i.e. with the 36 outcome variables this implies a significance threshold of 0.05/36 = 0.00139.

**Statistically significant effect at the α = 0.01 level after a Bonferroni correction for multiple comparisons, i.e. with the 36 outcome variables this implies a significance threshold of 0.01/36 = 0.00028.

The difference-in-difference analysis using the total sample did not result in any significant differences in health outcomes or cumulative resource use over time between the Salut and non-Salut areas (ATT estimates). Although the results suggest a change in a positive direction for most of the health outcomes (but not for resource use), we can note that the Programme did not have any effect on these outcomes for the mothers and children included in the analysis.

The longitudinal analysis showed significant improvements in pregnancy length and birth weight. These changes translate into mothers exposed to the Programme having experienced, on average, 6% (95% CI 3–9%) more full-term pregnancies and 2% (95% CI 0.03-3%) more babies with a birth weight ≥ 2500 g. For our sample, this translates into five additional mothers having full term pregnancies and one more child being born within normal weight range.

We observed a change in a positive direction for most other health outcomes and for resource use, although not statistically significant. We estimated the number needed to treat to prevent one pregnancy not reaching full term by dividing one by the absolute risk reduction between Salut and non-Salut (0.06); and the number needed to treat to prevent one baby not being born within normal weight range, by dividing one by the absolute risk reduction between Salut and non-Salut (0.02). Seventeen mothers would need to be exposed to the Salut Programme for one more pregnancy to reach full term and 50 mothers would need to be exposed for one more baby to be born within normal weight range.

### Costs

Table 4 shows the differences in cumulative costs related to health care service use and benefits related to care of sick child between Salut and non-Salut areas, for the period from birth until children completed five years of age. For the total sample, the difference-in-difference analyses showed that mothers in the Salut area incurred lower total costs (-$705) and lower inpatient costs (-$1,002), however higher outpatient care costs and higher benefits related to care of sick child than mothers in the non-Salut area. Nevertheless, these differences did not reach statistical significance. Children in Salut area incurred larger overall costs, although not significant, than children in the non-Salut area.

**Table 4:** Differences in costs between the Salut and the non-Salut areas, for the total sample and the longitudinal sample (in 2020 $US).

		Total sample (DinD)		Longitudinal sample (comparison of post measures only)	
Group	Outcome	Mean (95% CI)	p-value	Mean (95% CI)	p-value
Mothers	Inpatient care	-1,002 (-5,797, 3,793)	> 0.05	5,989 (-2,471, 14,449)	> 0.05
	Outpatient care	111 (-81, 304)	> 0.05	-483 (-1,217, 250)	> 0.05
	Care of sick child	186 (-225, 597)	> 0.05	-757 (-1,966, 453)	> 0.05
	Total	-705 (-5,552, 4,143)	> 0.05	1,685 (-14,008, 17,379)	> 0.05
Children	Inpatient care	2,775 (-2,213, 7,764)	> 0.05	-5,564 (-12,046, 918)	> 0.05
	Outpatient care	7 (-171, 185)	> 0.05	**-826 (-1,446, -206)**	**0.009**
	Total	2,782 (-2,604, 8,169)	> 0.05	-9,044 (-22,400, 4,311)	> 0.05
	Overall total	2,078 (-5,113, 9,269)	> 0.05	-8,073 (-34,807, 18,660)	> 0.05

The longitudinal analysis, in which post-measure values were compared for mothers who gave birth in both periods and remained living in the same geographical area over the analysis period, revealed an overall lower resource use for both mothers and children in Salut area. This was not true for inpatient care costs for mothers, which were larger than in the non-Salut area ($5,989). These differences did not reach statistical significance apart from outpatient care costs for children. Children in Salut area incurred significantly lower outpatient costs (-$826). Mean cost descriptives per mother per child, before matching, for the total sample and the longitudinal sample for both Salut and non-Salut areas are available in tables S3 and S4 in the Supplementary Appendix.

## Discussion

This study compared health and economic outcomes of the Salut Programme, an early intervention delivered universally through Swedish ordinary public services to parents and their children, compared with care-as-usual, from pregnancy until the child reached five years of age. The longitudinal analysis on mothers, who had given birth in both the pre- and post-measure periods, showed that those who had been exposed to the Salut Programme, had on average 6% more full term pregnancies and 2% more babies with a birth weight ≥ 2500 g, compared to mothers who had only care-as-usual. Savings were also incurred in terms of outpatient care related costs for children of mothers in the Salut area. One interpretation of these findings is that the multisectorial, family-centred approach of the Salut Programme achieved health gains as a health promotion early intervention and may lead to lower usage of outpatient healthcare.

Matching of the study samples was used to simulate a natural experiment and resulted in covariate balancing, which improved comparability between the groups. These methods used to estimate the differential impact of the Salut Programme on the outcomes do not require strong assumptions. Two analytical approaches were considered to account for confounding. Aware of the risk for residual confounding in the difference-in-difference analysis using the full sample, a longitudinal analysis using mothers who had given birth both in pre and post measure periods was conducted. In this analysis, each mother´s outcome value at pre-measure was further included as a confounder. This analysis, however, rested on a small sample size, and although many indicators point towards a favourable direction of positive effects of the Salut Programme, very few reached statistical significance. The small sample size could have contributed to underestimation of the differences between groups and may, thus, limit the generalizability of findings.

Pre term birth is associated with negative consequences on the child’s health and overall wellbeing [26]. Consequences such as neurodevelopmental disorders including cerebral palsy, visual- and hearing impairments, have all been linked to pre term birth which yield large disease and financial burden to children and their families as well as society as a whole. Other outcomes of relevance for future complications for the child are low birth weight and SGA, the latter essentially establishing a relationship between birth weight and pregnancy length. Evidence is controversial on the relative impact of birth weight and pregnancy length on the development of pre-term birth babies [27]. The presence of more full term pregnancies in the Salut arm could potentially explain the larger number of ≥ 2500 g babies and fewer babies born SGA.

Evaluations of early interventions delivered universally through ordinary public services, such as the Salut Programme, are scarce. To our knowledge, there are no studies evaluating long-term effects and costs of comparable programmes. Nevertheless, available evidence has confirmed that the range of early services, from pregnancy to early childhood, can successfully improve parents’ and children’s outcomes and generate benefits that can outweigh programme costs. A meta-analysis of 115 early interventions targeting children or parents of children from the prenatal period to age five demonstrated that most of the reviewed programmes had favourable effects on at least one child outcome, and those with an economic evaluation tended to show positive economic returns [7]. For example, the pooled effect of the studies included indicated the improvement in birth outcomes, which is in the line with our results. Another meta-analysis reported cost-standardized effect estimates from 10 randomized controlled trials for children aged 1.5–24 years, conducted in Denmark. These interventions showed significant effects relative to their costs, while interventions targeted at younger children tended to produce larger effects [28].

The most notable strength of this study was that analyses were based on real world data from national Swedish registers, some of which had been routinely collected from medical records, which reflects routine praxis and overcomes the constraints of controlled studies, including non-representative study populations and missing data [29]. However, a limitation is that we did not have access to data from the medical records of the child healthcare services, which contain vast information of interest (e.g. breastfeeding duration and the child’s growth trajectory). Also, register data on primary care resource use and medication consumption was not available, which possibly could have resulted in an underestimation of the true difference in healthcare resource use and related costs, given that a big chunk of resource use for this population could be within primary care. Additionally, we believe that it is important to explore what the resource use figures translate into money terms, regardless of significance of the results from the analysis of frequencies of resource use. It is a transparent approach in relation to publication bias, and provides decision makers with valuable information.

Outcomes for mothers and their children were chosen based on the aims of the Salut Programme and its expected impacts on mothers and children’s health and wellbeing, but also these were heavily dependent on the data available in the registers used. This poses an important limitation to the present work and the extent to which the analysis captured the full range of potential benefits of the intervention. An example of a relevant variable to include would have been, for instance, the use of snuff, which is today more prevalent than smoking among pregnant women, and which has similar negative effects on preterm birth and fetal growth to smoking [30, 31].

Allocation of mothers was done based on place of residence at childbirth, which was based on the assumption that mothers had the same place of residence at conception, delivery and during the first years of the child´s life. The information of any changes in residence was unavailable in our dataset, and thus the assumption on mothers’ and children’s allocation to interventions or control areas according to place of residence at childbirth might be a limitation.

The Salut Programme was integrated into routine practice and, during the long follow-up period, some of the Salut pre-measure groups and some of the non-Salut post-measure groups were exposed to the intervention components for pre-school aged children. In other words, differences in exposure between groups were not as strict for pre-school aged children and their mothers compared to the more intensive intervention components that were delivered during pregnancy and up to child age 1.5 years. This may have resulted in an underestimation of the differences between groups.

Another limitation had to do with missing Diagnosis Related Group weights for a few of the years included in the analysis, which prompted us to take a conservative approach and adopt a costing methodology based on average costs per patient for all inpatient and outpatient care. This approach, although conservative compared to a mixed costing approach, could have led to an under or over estimation of the true costs. It is important to note that most outcomes available in the registers and used in the present analysis were hard clinical outcomes or proxy outcomes to actual health status of mothers and children.

No measures of wellbeing and quality of life were available in the registers. Clinical outcomes might not be the most adequate outcomes for measuring the impacts of such complex and multi-sectorial early health promotion programmes. Other measures, including health promotion outcomes (e.g. healthy lifestyles), as well as health and social outcomes (e.g. quality of life and disability), could have better captured the impacts of the Salut Programme [32]. Measures on fathers’ health, wellbeing and consumption of resource use were also missing, and although the interventions also targeted fathers, no information on fathers was available, which is a limitation.

More studies with longer-term follow-ups are needed to determine whether early intervention impacts are sustained over time. The findings demonstrate that the Salut Programme had favourable effects on at least two health outcomes and yield savings in terms of child outpatient costs. Our previous evaluation of the Salut Programme over the first two years of the child´s life suggested that it might be good value for money. Hence, the current evidence supports the continuous investment in this early childhood programme as it improves outcomes among children and their mothers at a low investment cost up to five years. Investments in the early years is key to promoting lifelong health and wellbeing and can have impacts beyond health, such as increased educational attainment, and outcomes in adulthood, such as employment and earnings [7]. Making use of existing service structures to deliver such interventions can be an efficient way of reaching out to all eligible parents and children.

## Conclusions

The Salut Programme, as a health promotion early intervention for children and parents, achieved health gains in terms of more full-term pregnancies and more babies with a birth weight ≥ 2500 g, at a reasonable cost, and may lead to lower usage of outpatient care. Other indicators point towards positive effects, but the small sample size may have led to underestimation of true differences. The current findings support the continuous investment in this early childhood programme.

## Electronic supplementary material

Below is the link to the electronic supplementary material.

**Supplementary Appendix A**: **Table S1**. The intervention package within the Salut Programme targeting parents and their children from foetal life to 1.5 years of age. **Table S2**. Unit costs used in costing analysis (in 2020 $US) - average estimates from Cost per patient database. **Table S3**. Mean costs per mother per child related to resource use in the Salut and the non-Salut areas, before matching, for the total sample (in 2020 $US). **Table S4**. Mean costs per mother per child related to resource use in the Salut and the non-Salut areas, before matching, for the longitudinal sample (in 2020 $US).

## Data Availability

The data that support the findings of this study are available from the authors, but restrictions apply. The data were used under license for the current study, and so are not publicly available.
