# Supplementary material for: Health and economic outcomes of a universal early intervention for parents and children from birth to age five: evaluation of the Salut Programme using a natural experiment
Source: Cost Eff Resour Alloc. 2023 May 4;21:29. doi: 10.1186/s12962-023-00439-7 (PMC10158322; doi:10.1186/s12962-023-00439-7)
Supplement: Supplementary file 1 — Supplementary Appendix A: Table S1. The intervention package within the Salut Programme targeting parents and their children from foetal life to 1.5 years of age. Table S2. Unit costs used in costing analysis (in 2020 $US) - average estimates from Cost per patient database. Table S3. Mean costs per mother per child related to resource use in the Salut and the non-Salut areas, before matching, for the total sample (in 2020 $US). Table S4. Mean costs per mother per child related to resource use in the Salut and the non-Salut areas, before matching, for the longitudinal sample (in 2020 $US). [file 12962_2023_439_MOESM1_ESM.docx]

**Supplementary Appendix A**

**Table S1.** The intervention package within the Salut Programme targeting parents and their children from foetal life to 1½ years of age.

. From: [Sustainable practice change: Professionals' experiences with a multisectoral child health promotion programme in Sweden](https://bmchealthservres.biomedcentral.com/articles/10.1186/1472-6963-11-61)

| **Intervention** | **Antenatal care** | **Child health care** | **Dental service** | **Open pre-school** |
| --- | --- | --- | --- | --- |
| Motivational interviewing | ***** | ***** | ***** |  |
| Collaboration between involved sectors | ***** | ***** | ****** | ****** |
| Parent meetings | ***** | ***** | ****** | ****** |
| Health counselling focusing on life habits, mental health, domestic violence^1^, parent-child attachment, psychosocial health and parent relationships | ***** | ***** | ****** |  |
| Edinburgh Postnatal Depression Scale (EPDS) screening |  | ***** |  |  |
| Oral health screening at 12 months of age |  | ****** |  |  |
| "Mothers visit" at child age 8 months including screening for domestic violence |  | ****** |  |  |
| "Fathers visit" at child age 10 months with focus on fathers experiences of change in life situation |  | ****** |  |  |
| Questionnaires for health surveillance | ****** | ****** | ****** |  |
| Free dental health care visit for the pregnant woman and her partner |  |  | ****** |  |
| Activities to enhance early parent-child attachment, children's physical activity and linguistic development |  | ***** |  | ***** |
| Activities supporting parents to establish contacts with each other |  |  |  | ***** |
| Activities to promote healthy snacks/food and drinks |  |  |  | ***** |

* Strengthening or restructuring of existing interventions

** Newly developed interventions within the Salut Programme

^1^ Pregnant women and women recently given birth

**Table S2**. Unit costs used in costing analysis (in 2020 $US) - average estimates from Cost per patient database.

| **Year** | **Mean cost per episode** | |
| --- | --- | --- |
|  | **Inpatient care** | **Outpatient care** |
| 2004 | 3,633.73 | - |
| 2005 | 3,716.93 | - |
| 2006 | 3,715.20 | - |
| 2007 | 3,737.72 | - |
| 2008 | 3,659.49 | 235.79 |
| 2009 | 3,689.72 | 275.30 |
| 2010 | 3,748.80 | 268.11 |
| 2011 | 3,740.21 | 266.93 |
| 2012 | 3,702.53 | 290.26 |
| 2013 | 3,942.00 | 306.02 |
| 2014 | 4,100.53 | 319.45 |

**Table S3.** Mean costs per mother per child related to resource use in the Salut and the non-Salut areas, before matching, for the total sample (in 2020 $US).

|  | **Salut area^a^** | | **Non-Salut area^a^** | |
| --- | --- | --- | --- | --- |
|  | **pre^b^** | **post^b^** | **pre^b^** | **post^b^** |
|  | **Mean (SD)** | **Mean (SD)** | **Mean (SD)** | **Mean (SD)** |
| **Mothers** |  |  |  |  |
| Inpatient care | 25,116 (34,375) | 26,161 (61,130) | 23,288 (52,216) | 22,989 (61,162) |
| Outpatient care | 576 (1,005) | 1,593 (2,239) | 557 (991) | 1,384 (1,963) |
| Care of sick child | 2,356 (2,486) | 2,801 (4,779) | 2,353 (2,579) | 2,659 (3,471) |
| Total | 28,049 (34,720) | 30,555 (61, 711) | 26,198 (52,422) | 27,032 (61,937) |
|  |  |  |  |  |
| **Children** |  |  |  |  |
| Inpatient care | 9,433 (30,032) | 8,682 (48,091) | 11,093 (51,773) | 8,214 (52,610) |
| Outpatient care | 536 (858) | 1,695 (2,159) | 559 (908) | 1,723 (2,256) |
| Total | 9,969 (30,321) | 10,377 (49,236) | 11,652 (52,084) | 9,938 (53,762) |
|  |  |  |  |  |
| Overall total | 38,018 (49,092) | 40,932 (80,949) | 37, 850 (76,948) | 36,969 (83,478) |
| M – mean; SD – Standard deviation.  ^a^ Salut area – Geographical area in Västerbotten county where the Salut Programme was implemented from 2006 and onwards; non-Salut area – remaining part of Västerbotten county.  ^b^ Premeasure period 2000-2002; postmeasure period 2006-2008. | | | | |

**Table S4.** Mean costs per mother per child related to resource use in the Salut and the non-Salut areas, before matching, for the longitudinal sample (in 2020 $US).

|  | **Salut area^a^** | | **Non-Salut area^a^** | |
| --- | --- | --- | --- | --- |
|  | **pre^b^** | **post^b^** | **pre^b^** | **post^b^** |
|  | **Mean (SD)** | **Mean (SD)** | **Mean (SD)** | **Mean (SD)** |
| **Mothers** |  |  |  |  |
| Inpatient care | 25,876 (18,213) | 21,936 (33,047) | 30,230 (52,633) | 20,204 (36,745) |
| Outpatient care | 561 (719) | 1,368 (1,307) | 532 (852) | 1,464 (2,260) |
| Care of sick child | 2,031 (2,177) | 2,381 (2,383) | 2,033 (2,137) | 3,279 (3,890) |
| Total | 28,467 (18,516) | 25,685 (33,102) | 32,795 (52,667) | 24,947 (38,018) |
|  |  |  |  |  |
| **Children** |  |  |  |  |
| Inpatient care | 10,887 (29,971) | 4,082 (8,832) | 11,507 (50,124) | 11, 624 (73,065) |
| Outpatient care | 677 (1,347) | 1,338 (1,645) | 562 (851) | 1,737 (2,520) |
| Total | 11,564 (26,608) | 5,420 (9,361) | 12,069 (50,507) | 13,361 (74,655) |
|  |  |  |  |  |
| Overall total | 40,031 (39,127) | 31,105 (34,702) | 44,864 (74,211) | 38,308 (85,035) |
| M – mean; SD – Standard deviation.  ^a^ Salut area – Geographical area in Västerbotten county where the Salut Programme was implemented from 2006 and onwards; non-Salut area – remaining part of Västerbotten county.  ^b^ Premeasure period 2000-2002; postmeasure period 2006-2008. | | | | |
